# Developing a city-wide, community-engaged cancer disparities research agenda

**DOI:** 10.1007/s10552-024-01919-8

**Published:** 2024-09-28

**Authors:** Amy E. Leader, Yawei Song, Evelyn T. González, Thierry Fortune, Nilsa Graciani, Charnita Zeigler-Johnson, Karen Glanz

**Affiliations:** 1 Division of Population Science, Department of Medical Oncology, Thomas Jefferson University, 833 Chestnut Street, 11th Floor, Philadelphia, PA 19107 USA; 2 Sidney Kimmel Cancer Center, Thomas Jefferson University, Philadelphia, PA USA; 3 Fox Chase Cancer Center - Temple Health, Philadelphia, PA USA; 4 Philadelphia Department of Public Health, Philadelphia, PA USA; 5 Esperanza College of Eastern University, Philadelphia, PA USA; 6 Perelman School of Medicine and School of Nursing, University of Pennsylvania, Philadelphia, PA USA

**Keywords:** Cancer disparities, Stakeholder coalition, Cancer research, Community-engaged research

## Abstract

**Introduction:**

In response to high levels of cancer disparities in Philadelphia, PA, three NCI-designated clinical cancer centers formed Philadelphia Communities Conquering Cancer (PC3) to bring stakeholders together and establish infrastructure for future cancer reducing initiatives. The PC3 coalition aimed to develop a prioritized cancer disparities research agenda in order to align cancer center resources and research interests with the concerns of the community about cancer, and to ensure that initiatives were patient- and community-centered.

**Methods:**

Agenda development activities culminated in a city-wide cancer disparities conference. The conference, attended by 55 diverse stakeholders, was the venue for small group discussion sessions about cancer concerns related to prevention, early detection, treatment, survivorship, and quality of life. Sessions were guided by a moderator guide and were audiorecorded, transcribed, and analyzed by the PC3 leadership team. Results were reviewed and consensus was achieved with the help of PC3’s Stakeholder Advisory Committee.

**Results:**

Stakeholders identified four thematic areas as top priorities for cancer disparities research and action in Philadelphia: *communication* between patients, providers, and caregivers; *education* that reaches patients and community members with tailored and targeted information; *navigation* that assists people in finding and accessing the right cancer screening or treatment option for them; and *representation* that diversifies the workforce in clinics, cancer centers, and research offices.

**Conclusion:**

A community-informed, prioritized research agenda provides a road map for the three cancer centers to collaborate on future initiatives that are important to patients and stakeholders, to ultimately reduce the burden of cancer for all Philadelphians.

**Supplementary Information:**

The online version contains supplementary material available at 10.1007/s10552-024-01919-8.

## Background

### The burden of cancer in Philadelphia

Philadelphia is the sixth largest city in the United States, home to more than 1.5 million residents [1]. It is also one of the poorest large cities in the U.S., with 23% of the population living below the federal poverty line [2]. Philadelphia’s population is very diverse, with 40% of residents identifying as Black or African American, 16% identifying as Hispanic or Latino, and 8% identifying as Asian [1]. More than one quarter of Philadelphians are immigrants or have a foreign-born parent [3]. Seven percent of residents lack some form of health insurance [1].

Cancer is the second leading cause of death in Philadelphia [4]. Each year, more than 6,000 Philadelphians are diagnosed with cancer and roughly 3,000 die of the disease [5]. Incidence and mortality rates for cancer are some of the highest in the state of Pennsylvania [5]. Cancer burden is unequally distributed among communities in Philadelphia, with the highest burden seen in higher-poverty neighborhoods in North and West Philadelphia [6]. African American and Hispanic Philadelphians, which comprise more than half of residents, largely bear the burden of cancer disparities, with higher-than-expected rates of most cancers [7]. Cancer risk factors, including obesity, smoking, alcohol consumption, physical inactivity, poor diet, and environmental exposures, are disproportionately higher in Philadelphia as compared to national averages [4].

### The role of NCI-designated cancer centers in Philadelphia

Philadelphia is home to three NCI-designated clinical cancer centers (Abramson Cancer Center at the University of Pennsylvania, Sidney Kimmel Cancer Center at Thomas Jefferson University, and Fox Chase Cancer Center - Temple Health), spread across the city in different neighborhoods, all focused on reducing the cancer burden, treating cancer patients, and improving quality of life for cancer survivors. As part of NCI requirements for community outreach and engagement (COE), each cancer center is required to maintain a bidirectional relationship with the communities that they serve, working to disseminate and implement evidence-based interventions and guidelines, public education, and public health policy recommendations [8]. This bidirectional relationship between communities and cancer centers creates an infrastructure and promotes an understanding of cancer that is holistic and transdisciplinary, encompassing of different views and experiences, culturally sensitive, and reflective of mutual goals.

### Launching a city-wide cancer disparities coalition

Philadelphia is at the heart of each of the three clinical cancer centers’ communities, creating a natural synergy and starting point for a city-wide coalition and a prioritized research agenda to reduce cancer disparities. While city-wide cancer coalitions have existed in other cities [9, 10], and a cancer site-specific collaboration exists in Philadelphia [11], the city has lacked an organizational infrastructure for key stakeholders, including community-based partners, to tackle the burden of cancer across all communities, cancer types, and populations in Philadelphia. In 2022, with extramural funding shared among the three cancer centers, Philadelphia Communities Conquering Cancer (PC3) was launched to engage Philadelphians to reduce cancer disparities through community engagement, resource alignment, information sharing, research, and prevention.

### The need for a city-wide cancer disparities research agenda

The successful process of establishing PC3 has underscored the need for both agreed-upon priorities for disparities research and established structures to meaningfully engage Philadelphia stakeholders and community members. Early activities included launching a coalition website (www.phillycancercollab.org), convening a diverse Stakeholder Advisory Committee, and conducting eight listening sessions with 126 participants from a variety of community groups across the city. Details about the listening sessions, including the findings, can be found on the PC3 website. We then convened a city-wide cancer disparities conference in June 2023 that had a primary objective of developing Philadelphia’s first-ever community-engaged prioritized research agenda. The day-long conference, attended by community stakeholders, patient advocates, local government representatives, and COE leaders from the three cancer centers, allowed participants to reflect on and discuss key cancer concerns in their community, followed by interactive discussions to determine the prioritized research agenda. What follows below is a detailed explanation of the city-wide disparities conference, the results of the research agenda, and how PC3 plans to leverage the results going forward in future community-engaged cancer efforts.

## Methods

### Stakeholder engagement

A Stakeholder Advisory Committee (SAC) meets monthly and helps to guide all of the initiatives of PC3, including the city-wide disparities conference and the prioritized research agenda. It is led by a Community Chair and Co-Chair, both with longstanding ties to Philadelphia and to the cancer centers, and is comprised of nine members. The members were purposefully selected to represent diverse communities across Philadelphia including those who are Hispanic, African American, Asian, LGBTQ+, older adults, cancer patients and survivors, members of faith-based groups, and immigrants and refugees. During the conference planning phase, the SAC provided input on the agenda, suggested invited speakers, and shared names of potential attendees; on the day of the conference, several SAC members led breakout sessions and helped with gathering attendees’ thoughts about the prioritized research agenda.

### Conference attendees

We invited 83 people who represented diverse communities, organizations, and institutions across Philadelphia who had an interest in reducing cancer disparities. Of those, 55 attended, including eight members of the SAC, eleven from cancer patient advocacy organizations, eight from non-profit or community-based organizations primarily representing immigrant or underrepresented communities in Philadelphia, three from faith-based organizations, two from a local government health department, five from a local biotechnology company, and 18, including the conference organizers, from the COE programs of the three cancer centers.

### Conference agenda

The agenda was designed to provide attendees with background information, as well as sufficient time for discussion, to ultimately be able to contribute their ideas for the prioritized research agenda. Morning sessions began with a review of cancer trends and disparities data for Philadelphia, followed by a presentation on local initiatives from the American Cancer Society. The SAC Chair and Co-Chair shared findings from eight listening sessions that were held across the city to engage diverse groups of residents in understanding their concerns about cancer prevention, treatment, and survivorship. There was time in the agenda for people to share personal stories about cancer, as well as to provide thoughts about the presentations and ask questions to the panelists.

### Conference breakout sessions

The focal point of developing the prioritized research agenda were small breakout sessions with scripted discussion questions to elicit attendees’ thoughts about cancer disparities research priorities. The leadership team, informed by the SAC, developed a moderator guide that asked attendees to think about the most important research areas for the three cancer centers to focus on related to: cancer prevention and screening, diagnosing cancer and connections to care, cancer treatment and caregiving, and survivorship and quality of life. After going through each of these topic areas, the moderator guide asked, “Of all of the priorities that we’ve discussed here, what do you think is the GREATEST research priority?” The moderator guide is included in the Supplemental Appendix. Breakout sessions occurred during one hour over lunch and were individually moderated by SAC members. Many of the SAC members had experience moderating group discussions in their professional capacity; all SAC members were briefed ahead of time with the moderator guide and the goals for the session. Additionally, a member of the leadership team was available for questions or assistance while the sessions occurred. Session discussions included a notetaker in each group, were audiorecorded, and later professionally transcribed. Each of the five groups included between seven and nine conference attendees.

### Presentation of breakout session discussion highlights

At the conclusion of the breakout sessions, each group presented its findings to all attendees by summarizing their notetaker’s notes. Members of each group had a chance to add thoughts beyond the notetaker’s summary, and other attendees could comment on each group’s key findings. By the end of reporting across the six groups, a high-level prioritized research agenda consisting of four domains was taking shape.

### Analysis of breakout session discussions

The research agenda was further informed by the audiorecordings of the breakout sessions and the notetakers’ notes. Upon receipt, members of the leadership team reviewed the transcripts to confirm the preliminary results from the consensus conference as well as ensure that no major themes or trends were identified during the breakout sessions that were not reported during the discussion highlights. The four domains identified by the stakeholders at the conference remained unchanged. Final consensus was achieved by sharing and discussing the results with the SAC members, who also provided insight into community priorities and perspectives.

## Results

### City-wide, community-engaged cancer disparities research agenda

The discussions held at the city-wide cancer disparities conference led to the following four thematic areas being prioritized by stakeholders as the most important research foci to contribute to reducing cancer disparities:

o Communication: Identifying and evaluating strategies to improve communication between healthcare providers, patients, and caregivers about cancer-related health decisions, especially for older adults
o Education: Identifying novel ways to reach patients and community members with tailored and targeted information about cancer prevention, screening, and treatment
o Navigation: Understanding the most effective ways to help patients identify and access appropriate screening and cancer care services
o Representation: Investing in workforce and training programs to increase the diversity of the workforce in clinics, cancer centers, and research offices, thereby improving the public and patients’ trust in cancer research and health care services

## Discussion

PC3, and the city-wide cancer disparities research agenda, are responses to the high burden of cancer disparities in Philadelphia blended with an effort to leverage the resources of three NCI-designated cancer centers and synergize research and intervention efforts in the future. This collective impact approach can serve as a model for other cancer centers in close proximity with overlapping cancer centers, to work together on common priorities. Indeed, a recent analysis of cancer center catchment areas found that roughly one-third of U.S. counties are in the primary catchment area of more than one cancer center [12]. The COE initiatives in each cancer center are poised to lead this collaborative work. The ability of cancer centers to work together, with the partnership of stakeholders, to implement community-important research priorities is paramount to overcoming the disproportionate burden of cancer in the U.S.

One of the four research priorities identified by stakeholders was *communication*, or improving the quality of discussions between the health care team and patients, families, and caregivers. Research has shown that provider recommendation, shared decision making, and high quality communication are important in a patient’s decision to screen for cancer and lead to higher screening uptake [13]. Communication may be even more important when navigating the complexities of cancer care, where decisions about treatment, clinical trials, and care management are of utmost importance. While many patients, especially those who are older, report difficulties with provider communication [14], patients who are immigrants or from racial and ethnic minority groups report some of the highest levels of communication difficulties [15]; reduced quality of communication between language discordant providers and patients has actually been shown to negatively impact cancer outcomes [16]. Because of this, many interventions, including those being proposed by PC3, aim to improve provider communication or patient engagement in health care decisions [17]. Additionally, national cancer organizations have proposed guidelines on how effectively communicate with patients [18].

A second research priority was *education*, or identifying novel ways to reach patients and community members with tailored and targeted information about cancer prevention, screening, and treatment. Undoubtedly, the intricacies of cancer care are difficult for many patients to understand [19]; the more recent integration of genetics, genomics, and precision medicine into cancer treatment decisions has made the information even more complex [20]. Separately, many patients and caregivers use the Internet to find health information [21], suspecting themselves to misinformation and disinformation about cancer risk or outcomes [22]. Data from a nationally representative survey recently found that there is a subset of individuals, about 15% of the population, that do not believe that the health information on the Internet is misleading [23]. Interventions that increase the health and digital literacy of people, particularly those with lower educational levels, is needed. Additionally, interventions that have provided targeted or tailored patient information across the cancer continuum have shown promise [24]; ensuring that these types of interventions exist in Philadelphia, as well as developing new interventions to educate patients and communities, is important.

A third cancer disparities research priority was *navigation*, or support for identifying the most effective ways to help patients find and access appropriate screening and cancer care services. Patient navigation has been shown to be effective in many contexts in cancer [25], from helping people access appropriate screening services [26], to helping patients receive an accurate diagnosis of cancer and begin treatment [27]. Navigation interventions may be particularly effective when pairing patients with navigators who have similar lived experiences, such as in minority or immigrant communities [28]. Research is still emerging about the best type of navigator for various situations, with some utilizing nurse-based navigation programs for newly diagnosed patients and others utilizing community health workers to navigate patients to cancer screening programs [29]. In any context, determining the best way to get people to the services that they need, in a way that is relatable, is important to reducing cancer disparities in Philadelphia.

The final research priority identified by stakeholders was *representation*, or investing in workforce and training programs to increase the diversity of the workforce in clinics, cancer centers, and research offices, thereby improving the public and patients’ trust in cancer research and health care services. Lack of representation in the cancer workforce, due to structural and societal barriers [30], is a pressing issue that has very likely contributed to longstanding cancer disparities. The current oncology workforce, whether measured by physician demographics [31] or those who work in nursing and cancer research [32], rarely matches the demographics of the catchment area and patient population that is being served. This creates a division between the patients and the providers and can lead to mistrust. Interventions to diversify staff through programs meant to mentor students and trainees in the early stages of their careers shows substantial promise [33]. Understanding how PC3 can undertake or contribute to efforts to increase representation in local cancer center research staff could ultimately lead to increased participation of underrepresented communities in cancer clinical trials [34]. Interestingly, one of the next initiatives of PC3 is to train community members to be local cancer research advocates.

The work described here is a culmination of establishing a city-wide cancer coalition, convening a diverse Stakeholder Advisory Committee, and hosting a cancer disparities conference to determine Philadelphia’s first-ever community-engaged cancer disparities research agenda. Other cancer centers can and should take advantage of these processes to replicate similar initiatives in their catchment areas. Stakeholders identified broad themes for priority research areas, such as education and navigation, rather than focusing on a specific type of cancer or stage of cancer research. A similar approach involving professional experts identified themes of patient navigation, community engagement, and healthcare system changes as priorities for addressing cancer disparities in the U.S. [35]. These broader, theme-based agendas allow for a certain amount of flexibility when implementing research to align with the agenda, as investigators can determine which cancer types or stage along the cancer continuum is best suited to the theme. Since the hosting of the consensus conference, the three cancer centers have trained 18 community members in the fundamentals of community-based cancer research, for future research collaborations. Some PC3 SAC members and stakeholders will be involved in newly secured grant funding across the cancer centers to improve communication between providers and older adult cancer patients, one of the priority research agenda themes. All of this work will continue in partnership with patients, stakeholders, and community members to ensure that it is reflective of their needs and circumstances. It is the hope of all of those involved in PC3 that this community-engaged, prioritized research agenda contributes to a significant reduction of the burden of cancer in Philadelphia.

## Supplementary Information

Below is the link to the electronic supplementary material.

Supplementary file1 (DOCX 21 KB)

## Data Availability

Data that was analyzed in this manuscript is available upon request from the corresponding author.
