# Supplementary material for: Developing a city-wide, community-engaged cancer disparities research agenda
Source: Cancer Causes Control. 2024 Sep 28;36(1):45–50. doi: 10.1007/s10552-024-01919-8 (PMC11762217; doi:10.1007/s10552-024-01919-8)
Supplement: Supplementary file 1 — Supplementary file1 (DOCX 21 KB) [file 10552_2024_1919_MOESM1_ESM.docx]

| **Philadelphia Cancer Disparities Consensus Conference**  **Breakout Session Moderator Guide** | | | |
| --- | --- | --- | --- |
|  | **Tips for Moderating the Discussion** | | |
|  | - Ground rules:   - Confidentiality – What is said here stays here!   - Cell phones on vibrate or silent and stored away. Please take calls outside of the room.   - Please be honest with your responses even if you think your comments may be considered controversial.   - Respect others’ opinions and viewpoints.   - One person talking at a time.   - No side-bar chatting, it’s too hard to hear (suggested reversing order w/ above point)   - Agree to disagree.   - Speak up clearly, as the discussion is being recorded and we want to capture everything   - FOR THE MODERATOR: Keep in mind that we are hoping to come up with RESEARCH priorities in the end; we don’t want to put people off who may not feel confident speaking about research. See the point **highlighted in blue** and do what you can to probe for research prioirites! | | |
| **Time** | **Discussion Questions** | | |
| **Start** | | Cumulative Time | |
| 5 minutes | Introduction:  The purpose of today’s meeting is to create an agenda of cancer-related priorities for Philadelphia – ones that can be addressed by cancer researchers at Penn, Fox Chase, and Jefferson.  The goal of this discussion over the next hour is to talk about what you think are the most important cancer-related issues in Philadelphia, based on your own experiences and from what you’ve heard today.  As the moderator, I will be asking you a series of questions to guide our discussion so we can begin to create a roadmap of cancer related research priorities for Philadelphia. We will be recording this session and there is also a note-taker in our room, to capture some themes and discussions.  After this session ends, we will be reporting back each group’s top priorities related to cancer, for group-wide voting on Philadelphia’s priority cancer and cancer research agenda. XX will be taking notes and giving our group’s report (or – we will ask for a volunteer to give our report)  If time permits, I’d like to ask you about how you’d like to see the Cancer Coalition function in the future, and what you would like to see from it. | 00:05 | |
| 3 minutes | **Q1: Let’s quickly go around the room and introduce ourselves – just your name and the organization you represent.** | 00:08 | |
| 5 minutes | **Q2: From the things you’ve heard this far today, did anything stand out or surprise you? What was that, and why?** | 00:13 | |
|  | | | |
|  | **Now we’re going to discuss what we think are the most pressing issues in Philadelphia related to cancer. I’m going to break it down into: cancer prevention and screening; diagnosis and connection to cancer care; cancer treatment and caregiving; survivorship and quality of life. Ultimately, we want to think of these things as areas for future research.** | | |
|  | | | |
| 8 minutes | **Q3: When you think about *cancer prevention or screening*, what do you think is most important for the cancer centers in Philadelphia to focus on?**  Examples might be:   - Cancer prevention: reducing risk factors like tobacco use - Cancer screening: helping more people get cancer screening | 00:21 | |
|  | | | |
| 8 minutes | **Q4:** **When you think about *diagnosing cancer and connection to care*, what do you think is most important for the cancer centers in Philadelphia to focus on?**  Examples might be:   - Diagnosing cancer: ensuring that people who might have cancer get diagnosed as quickly as possible - Connection to care: helping people who need follow-up care navigate the health care system | | 00:29 |
|  | | | |
| 8 minutes | **Q5: When you think about *cancer treatment and caregiving*, what do you think is most important for the cancer centers in Philadelphia to focus on?**  Examples might be:   - Cancer treatment: access to clinical trials - Caregiving: reducing caregiver burn-out | | 00:37 |
| 8 minutes | **Q6:** **When you think about *survivorship and quality of life*, what do you think is most important for the cancer centers in Philadelphia to focus on?**  Examples might be:   - Survivorship: preventing cancer recurrence (cancer coming back) - Quality of Life: helping cancer patients and survivors live the life they desire | | 00:45 |
|  | | | |
| 8 minutes | **Q7: Of all of the priorities that we’ve discussed here, what do you think is the GREATEST *research* priority? Why?** | | 00:53 |
|  |  | |  |
| If time permits | **Q8: For the newly formed Philadelphia Communities Conquering Cancer (PC3), what would you like to see happen with the coalition?**  Probe: Working groups on particular cancer-related topics, regular meetings or an annual meeting, updates and opportunities to participate in cancer research, distribution of cancer related information or resources for communities, etc….. | | 00:60 |
